# DUSP2 inhibits the progression of lupus nephritis in mice by regulating the STAT3 pathway

**DOI:** 10.1515/biol-2022-0649

**Published:** 2023-07-17

**Authors:** Xingzhong Liu, Jie Chen, Lu Liu

**Affiliations:** Department of Clinical Laboratory, Affiliated Sichuan Provincial Rehabilitation Hospital of Chengdu University of TCM, Sichuan, Chengdu Province, 611135, China; Department of Nephrology, Wuhan Third Hospital, 241 Pengliuyang Road, Wuhan, Hubei Province, 430074, China; Pediatric Clinic, Wuhan Third Hospital, Wuhan, Hubei Province, 430074, China

**Keywords:** lupus nephritis, DUSP2, inflammation, kidney injury, STAT3

## Abstract

One of the most severe side effects of systemic lupus erythematosus (SLE) is lupus nephritis (LN). To search for potential therapeutic targets in SLE is crucial for the progression of SLE. In this study, we selected C57BL/6J mice as controls and MRL/lpr mice as an LN model and obtained dual specificity phosphatase 2 (DUSP2)-overexpressed mice by injecting AAV-DUSP2 plasmid into the tail vein. Then, proteinuria, urea nitrogen, dsDNA and TNF-α, IL-6, and IL-1β levels were measured in each group of mice. In addition, renal histopathological damage was assessed by hematoxylin–eosin. Finally, STAT3 phosphorylation levels were detected by Western blot assay. The results showed that DUSP2 could reduce proteinuria, urea nitrogen, dsDNA and TNF-α, IL-6, and IL-1β levels and improve renal tissue injury in mice with LN. Mechanistically, DUSP2 inhibited STAT3 phosphorylation. These results demonstrated that DUSP2 played a role in ameliorating LN, which provided potential targets for LN research.

## Graphical abstract

**Figure:**
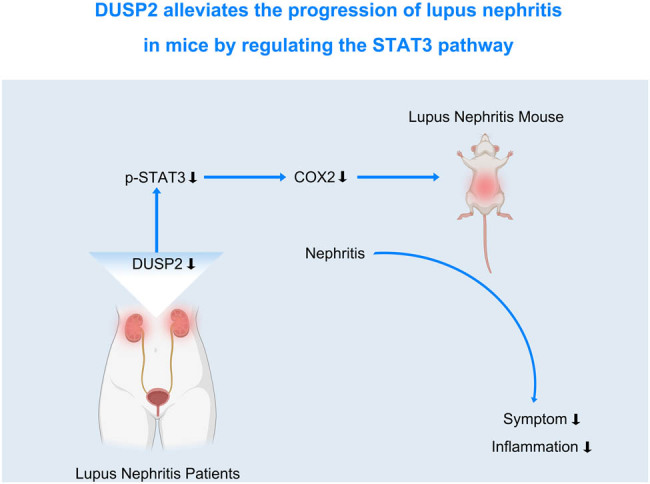

## 1 Introduction

Systemic lupus erythematosus (SLE) is an autoimmune disease that is more prevalent in women than in men and usually happens in young adulthood. It is thought to be caused by an over-activation of the autoimmune system attacking its own tissues and organs, with kidney tissue being the most susceptible organ [1]. About 50% of SLE patients have clinical manifestations of kidney damage, called lupus nephritis (LN), which mainly manifests as elevated inflammatory factors, proteinuria, and impaired renal function [2]. Due to the complex pathogenesis of LN, there are no effective drugs to cure LN for the time being. Therefore, it is crucial to find potential therapeutic targets for the treatment of LN.

There are 25 phosphatases in the dual specificity phosphatase (DUSP) family, all of which dephosphorylate their substrates at threonine/serine residues and/or tyrosine residues [3]. DUSP family members are involved in autoimmune disorders. For instance, the expression of DUSP22 is downregulated in SLE, and the downregulation of DUSP22 is associated with poor prognosis of LN patients [4]. Dual specificity phosphatase 2 (DUSP2), a member of the DUSP family, is also known as activated cell phosphatase 1 (PAC-1). DUSP2 has been reported to be involved in human autoimmune diseases, and transcript levels of DUSP2 are reduced in peripheral blood mononuclear cells from patients with ulcerative colitis [5]. Furthermore, previous studies have shown that DUSP2 deficiency exacerbated tubular injury and the progression of acute kidney injury, whereas DUSP2 overexpression prevented acute kidney injury [6].

However, the role of DUSP2 in LN and related mechanisms are not clear. In this study, we determined the expression of DUSP2 in human tissue samples and mice with LN and mice with DUSP2 transfection. We found that DUSP2 could regulate STAT3 phosphorylation to improve LN renal inflammation and renal function. In conclusion, our study provides potential targets for treating LN.

## 2 Methods

### 2.1 Clinical samples

For diagnostic purposes, kidney tissue was obtained from 20 LN (category IV) patients. The tumor-adjacent tissues of 10 patients who underwent renal tumor resection were selected as normal tissues. This study was approved by the Ethics Committee of Wuhan Third Hospital. All patients have given informed consent.


**Informed consent:** Informed consent has been obtained from all individuals included in this study.
**Ethical approval:** The research related to human use has been complied with all the relevant national regulations, institutional policies and in accordance with the tenets of the Helsinki Declaration, and has been approved by Ethics Committee of Wuhan Third Hospital.

### 2.2 Animals

MRL/lpr mice and C57BL/6J mice were purchased from Shanghai SLAC Laboratory Animal Co., Ltd. MRL/lpr mice are spontaneous lupus erythematosus mice with similar symptoms to human lupus erythematosus. All animals were housed in a pathogen-free environment. All animal studies were approved by the Ethics Committee of Wuhan Third Hospital and were conducted in accordance with the National Institutes of Health Guidelines for the Care and Use of Animals. The animals were divided into three groups: C57BL/6J normal mice as the control group (*n* = 6), MRL/lpr mice injected with empty vector via tail vein as the vector group (*n* = 6), and AAV-DUSP2 plasmid injected mice as the DUSP2 group (*n* = 6).


**Ethical approval:** The research related to animal use has been complied with all the relevant national regulations and institutional policies for the careand use of animals, and has been approved by the Ethics Committee of Wuhan Third Hospital.

### 2.3 Assessment of proteinuria level

Mice were placed in metabolic cages at 12 weeks and 24 h urine was collected every 2 weeks, and proteinuria was measured by Multistix 10SG reagent strips (Bayer Healthcare, IN, USA) [7]. The proteinuria scores were calculated as described previously [8].

### 2.4 Assessment of urea nitrogen and dsDNA levels

To collect blood from mice, the clot was kept naturally at room temperature for 30 min and then centrifuged at 1,000 × *g* for about 15 min. At last, the supernatant was collected, and the serum BUN content was measured by a BUN assay kit (Qindao Jisskang Biotechnology Co., Ltd.). Mouse serum dsDNA levels were measured using an anti-mouse dsDNA ELISA kit (Shanghai Fusheng Industrial Co., Ltd.).

### 2.5 Assessment of serum inflammatory cytokine levels

The serum of mice was collected, and the serum TNF-α, IL-6, and IL-1β (Shanghai Enzyme-linked Biotechnology Co., Ltd.) levels were measured according to the manufacturer’s instructions.

### 2.6 Kidney histopathological evaluation

The mice in each group were euthanized, and kidney tissues were obtained and fixed in 10% formaldehyde solution and then processed by dehydration and transparency steps. Finally, the kidney tissues were embedded in paraffin blocks and stained with eosin–hematoxylin staining solution [9].

### 2.7 qRT-PCR

Total RNA was extracted from kidney tissue using the TRIzol reagent (Invitrogen, USA). Total RNA was reversely transcribed into cDNA using SuperScript II (Invitrogen) reverse transcriptase [10]. qRT-PCR analysis was performed using the SYBR green method. Relative expression of target mRNA was calculated by the 2^−ΔΔCt^ method. The primer sequences are as follows: IL-6: forward primer AGATCTACTCGGCAAACC, reverse primer CGTAGAGAACAACATAAGTCAG; IL-1β: forward primer TGACCTGGGCTGTCCTGATG, reverse primer GGTGCTCATGTCCCTCATCCTG; and TNF-α: forward primer AGGCTGCCCCGACTACGT, reverse primer GACTTTCTCTGGTATGAGATAGCAAA.

### 2.8 Western bolt

Kidney tissues were cut into small pieces and ground into powder by adding liquid nitrogen. Then, RIPA lysis solution was added and lysed thoroughly on ice, and protein concentration was determined by BCA kit. Different groups of samples containing equal amounts of proteins were electrophoresed by 10% SDS-PAGE, and the protein bands were transferred to PVDF membranes. The membrane was blocked by 5% skim milk and incubated with DUSP2 (Solarbio, K009050P, 1:1,000, China), p-STAT3 (Beyotime, AF5941, 1:1,000, China), STAT3 (Beyotime, AF1492, 1:1,000), COX2 (GeneTex, GTX60935, 1:1,000, USA), and GAPDH (GeneTex, GTX100118, 1:5,000) antibodies overnight at 4°C in a refrigerator. The next day, the PVDF membrane was washed with TBST and incubated with HRP-labeled IgG (Abcam, ab205718, 1:10,000, UK).

### 2.9 Statistical analysis

Data were analyzed using SPSS version 20.0, and all data were expressed as mean ± standard deviation. Student’s *t*-test was used for comparison between two groups, and one-way ANOVA was used for comparison among multiple groups. *P* < 0.05 was considered to indicate a statistically significant difference.

## 3 Results

### 3.1 Low expression of DUSP2 in LN

**Figure 1:**
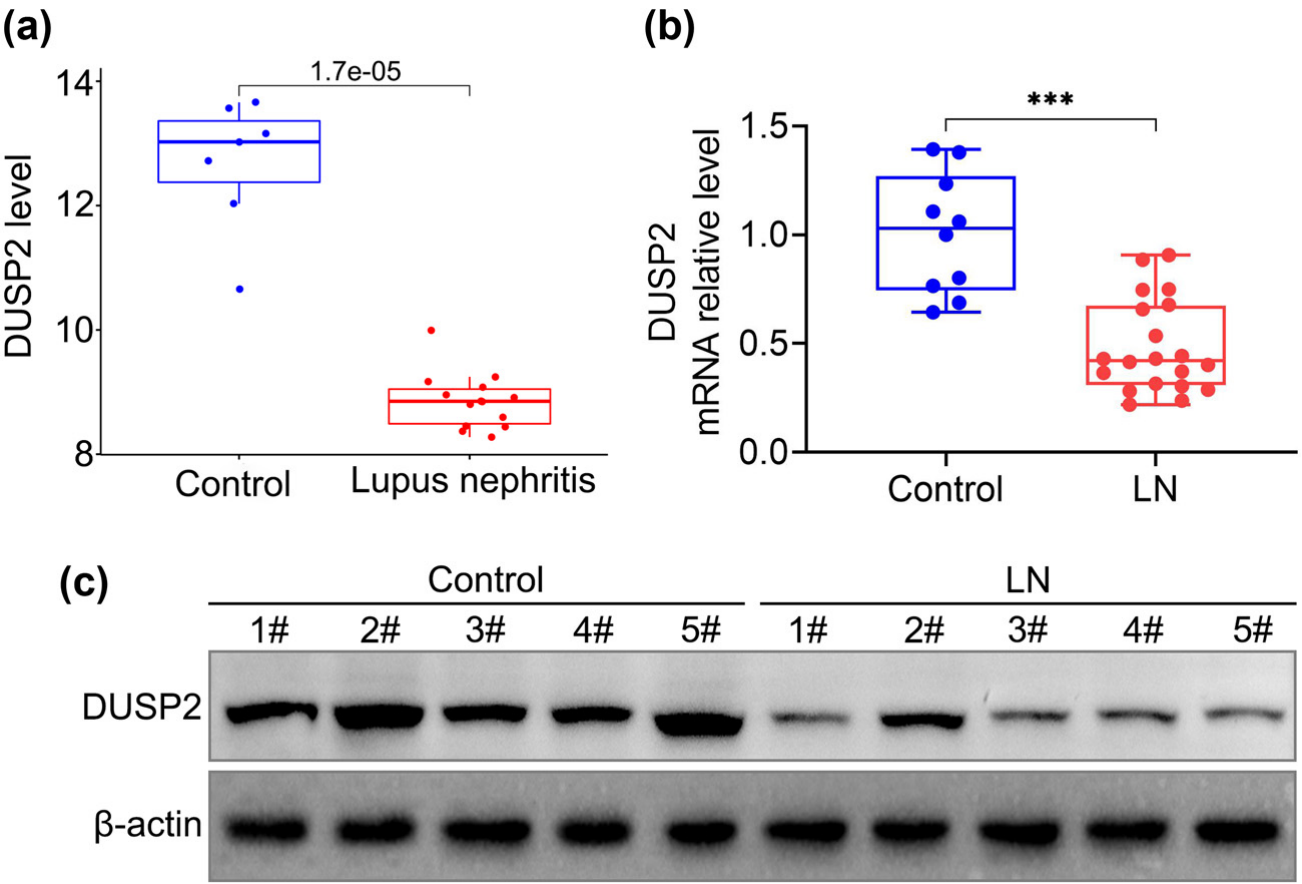
Low expression of DUSP2 in LN. (a) Expression of DUSP2 in normal and LN kidney tissues obtained from GSE112943 expression profile. (b) The mRNA expression levels of DUSP2 in normal and LN kidney tissues. (c) Protein expression levels of DUSP2 in normal and LN renal tissues. ^***^ *P* < 0.001 vs control.

We first analyzed the expression of DUSP2 in LN and normal kidneys from the GSE112943 expression profile and found that the expression of DUSP2 was significantly lower in kidney samples from patients with LN than in normal kidney tissue (Figure 1a). In addition, we examined the expression of DUSP2 in the renal tissues of patients with LN from our hospital and found that both mRNA (Figure 1b) and protein (Figure 1c) expression of DUSP2 were significantly decreased in the renal tissues of LN patients.

### 3.2 DUSP2 ameliorates renal tissue lesions in mice with LN

**Figure 2:**
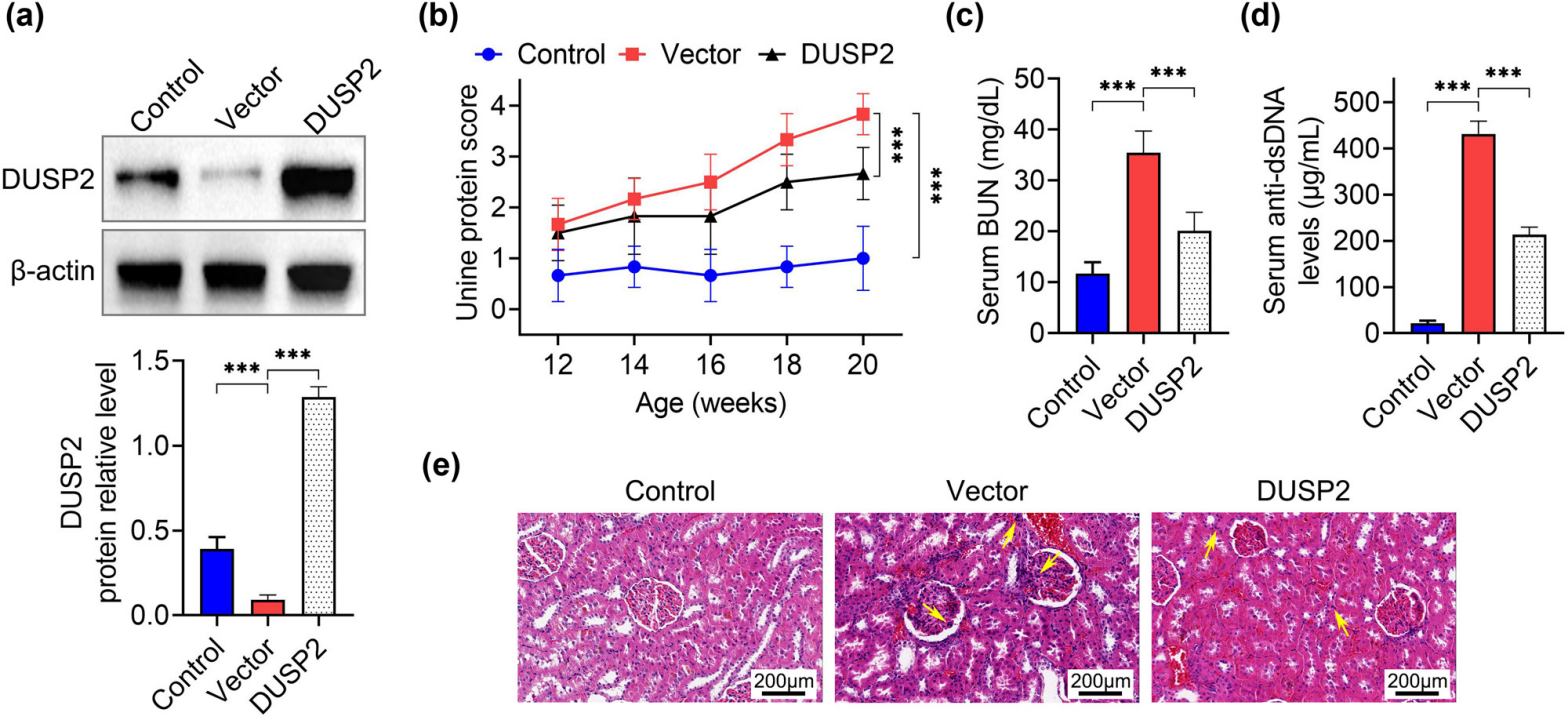
DUSP2 ameliorates renal tissue lesions in mice with LN. (a) Expression levels of DUSP2 protein in kidney tissues of various groups of mice. (b) Changes in proteinuria levels of mice in each group from 12 to 20 weeks. (c) Serum urea nitrogen levels in all groups of mice. (d) Serum dsDNA levels in each group of mice. (e) Renal histopathology of mice in each group ^***^ *P* < 0.001 vs vector.

To investigate whether DUSP2 plays a role in LN, we transfected DUSP2 overexpression plasmids into LN mice and found that DUSP2 expression was significantly increased in kidney tissues of mice transfected with DUSP2 (Figure 2a), indicating a successful transfection effect. We then examined the proteinuria levels in mice and found that the proteinuria levels in vector group mice were gradually increased during 12–20 weeks compared to the control group, while the proteinuria levels in the DUSP2 group were significantly decreased (Figure 2b). We also examined serum urea nitrogen and dsDNA levels in mice and found that serum BUN and dsDNA levels were significantly elevated in the vector group, while BUN (Figure 2c) and dsDNA (Figure 2d) levels were significantly decreased in DUSP2 group. Finally, we performed HE staining on the kidney tissues of mice and found that the kidney tissues of mice in the vector group showed neutrophil infiltration, and other pathological features of kidney disease, and the kidney tissue lesions of mice in the DUSP2 group were improved compared with those in the vector group (Figure 2e).

### 3.3 DUSP2 alleviates kidney tissue inflammation in mice with LN

**Figure 3:**
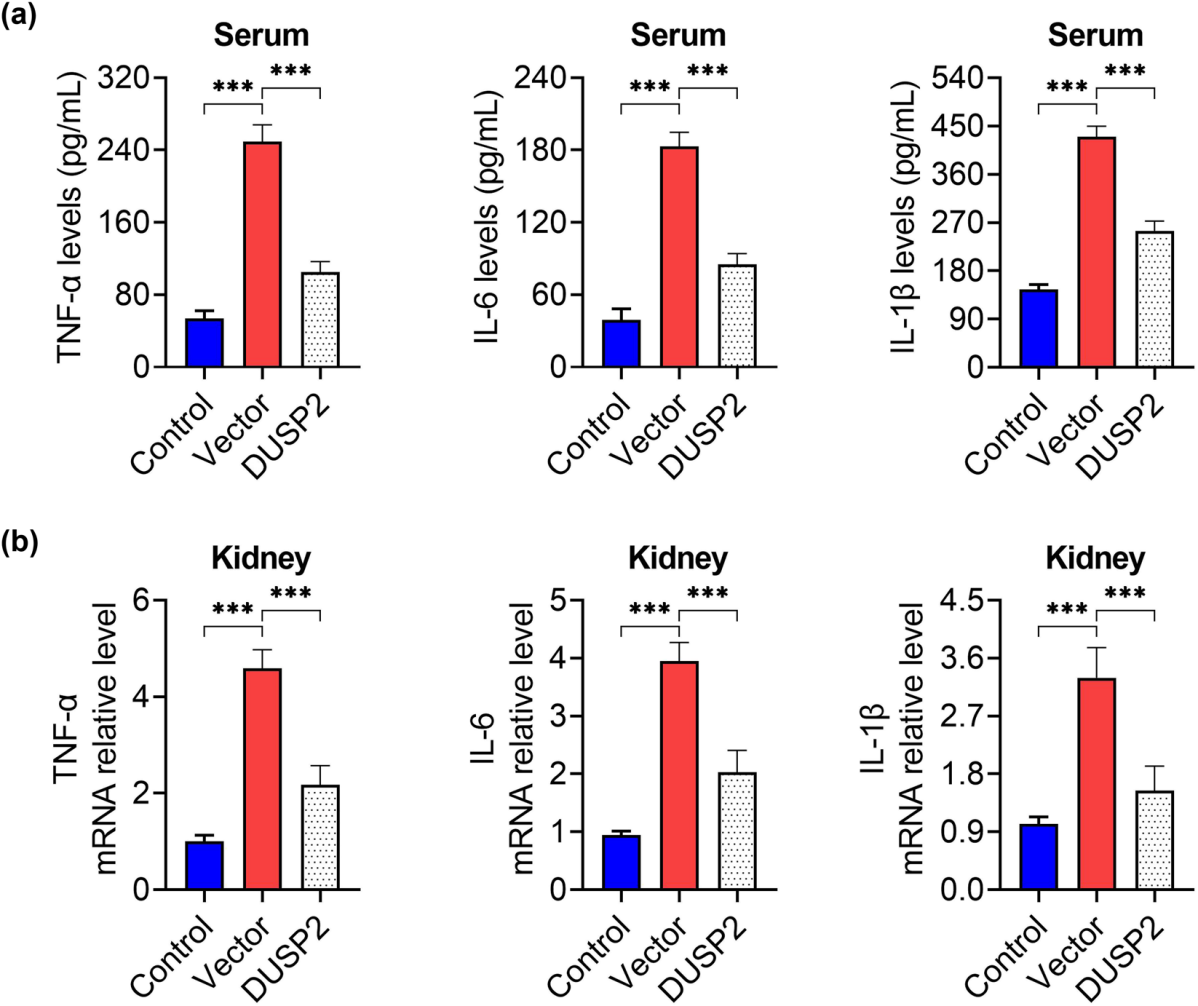
DUSP2 reduces kidney tissue inflammation in mice with LN. (a) Serum TNF-α, IL-6, IL-1β content of mice in each group. (b) Expression of TNF-α, IL-6, IL-1β mRNA in kidney tissue of mice in each group. ^***^ *P* < 0.001 vs vector.

To test whether DUSP2 reduces the inflammatory response in LN mice, we measured the levels of TNF-α, IL-6, and IL-1β in serum and kidney tissues by ELISA and PCR. The result revealed that the levels of TNF-α, IL-6, and IL-1β in serum (Figure 3a) and kidney tissues (Figure 3b) of mice in the vector group were increased, while the levels of the above inflammatory factors were significantly decreased after transfection of DUSP2.

### 3.4 DUSP2 inhibits STAT3 activation

**Figure 4:**
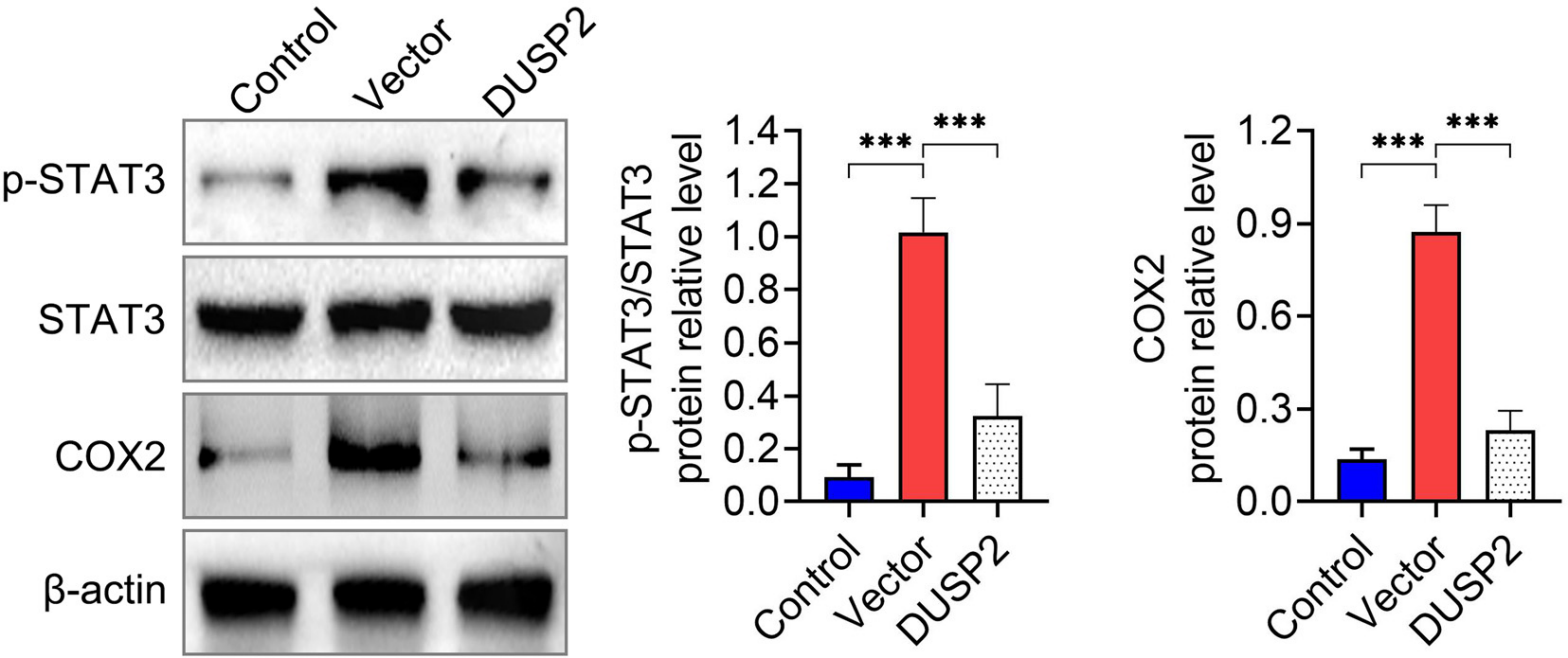
DUSP2 inhibits STAT3 activation. Expression of p-STAT3 and COX2 protein in kidney tissue of mice in each group. ^***^ *P* < 0.001 vs vector.

We examined the phosphorylation level of STAT3 and the expression of COX2, the downstream target of STAT3, in the kidney tissues of LN mice by western blot assay. We found that the expression of p-STAT3 and COX2 was increased in the kidney tissues of mice in the vector group, while the levels of p-STAT3 and COX2 were decreased in the kidney tissues of mice in the DUSP2 group (Figure 4). This indicated that DUSP2 inhibited the activation of STAT3.

## 4 Discussion

Previous studies have shown a protective effect of DUSP2 against acute kidney injury, but the role of DUSP2 in the autoimmune disease LN is unclear. Here, we chose MRL/lpr mice as a model because MRL/lpr mice exhibit various features such as nephritis, autoantibody production, and splenomegaly, which are similar to those of human lupus erythematosus [11]. In addition, MRL/lpr mice were transfected with DUSP2 overexpression plasmid and the proteinuria level in each group of mice was observed from 12 to 20 weeks and found that DUSP2 could reduce the proteinuria level in MRL/lpr mice.

BUN is one of the clinical indicators used to assess renal function. Kidney disease or failure is related to increased BUN levels [12]. Patients with SLE have a significant release of DNA-containing nucleosomes into their circulatory system, which results in the development of immune complexes including anti-dsDNA autoantibodies. After being deposited in the kidney, immune complexes induced a series of events that leads to renal inflammation and the production of chemokines, cytokines, and complement [13]. dsDNA antibody has a very specific diagnostic value for SLE. If this antibody is elevated, it means that the lupus disease is not fully controlled and needs to be treated actively. As lupus erythematosus disease is controlled, the level of dsDNA will gradually decline. Our findings suggested that DUSP2 restores impaired renal function in lupus mice by reducing BUN levels. Furthermore, DUSP2 also reduced dsDNA levels in MRL/lpr mice.

Immune dysfunction caused by unbalanced cytokine production also mediates tissue inflammation and harms organs. Inflammatory cytokines including IL-1β [14], IL-6 [15], and TNF-α [16] have been recognized as significant contributors to SLE. The severity and activity of glomerulonephritis are correlated with the expression of these cytokines [17]. Our results showed that DUSP2 reduced the levels of IL-1β, IL-6, and TNF-α in serum and renal tissues of MRL/lpr mice, demonstrating that DUSP2 improved the inflammatory response in LN.

Signal transducer and activator of transcription 3 (STAT3) is essential for controlling innate and adaptive immunological responses, as well as inflammation. LN results in abnormal STAT3 activation (phosphorylation). SLE T cells can respond to chemokines and move more quickly *in vitro* when STAT3 is phosphorylated [18]. The onset of LN has been found to be delayed by STAT3 inhibition [19]. In addition, cyclooxygenase 2 (COX2) expression was upregulated in LN [20], and low-dose COX-2 inhibitor celecoxib has ameliorative effects in lupus mice [21]. COX2 also acted as a downstream target of STAT3. DUSP2 is associated with STAT3 and attenuates its activity by inhibiting STAT3 phosphorylation [5]. Our results showed that DUSP2 decreased STAT3 phosphorylation levels and COX2 levels in kidney tissue of MRL/lpr mice, indicating that DUSP2 inhibited STAT3 phosphorylation in LN.

In conclusion, we demonstrated that the expression level of DUSP2 was decreased in LN for the first time. DUSP2 ameliorated LN and suppressed inflammation by inhibiting STAT3 phosphorylation levels. Our study provides promising potential targets for treating LN.
